# Extracellular DNA, hyaluronic acid, HIF pathways, and LncRNAs as predictive biomarkers of severe COVID-19

**DOI:** 10.1186/s12985-025-02886-5

**Published:** 2025-07-23

**Authors:** Evgen Dubrovskyi, Tetiana Drevytska, Alla Portnychenko, Victor Dosenko, Iryna Halabitska, Pavlo Petakh, Denis E. Kainov, Oleksandr Kamyshnyi

**Affiliations:** 1 Bogomoletz Institute of Physiology, National Academy of Science, Kyiv, Ukraine; 2 Department of Therapy and Family Medicine, I. Horbachevsky Ternopil National Medical University, Ternopil, 46001 Ukraine; 3 Department of Biochemistry and Pharmacology, Uzhhorod National University, Uzhhorod, Ukraine; 4 Department of Clinical and Molecular Medicine (IKOM), Norwegian University of Science and Technology, Trondheim, 7028 Norway; 5 Department of Microbiology, Virology, and Immunology, I. Horbachevsky Ternopil National Medical University, Ternopil, Ukraine

**Keywords:** COVID-19, SARS-CoV-2, NETosis, Extracellular DNA (cfDNA), Hyaluronic acid (HA), Hypoxia-inducible factors (HIF-1α, HIF-2α, HIF-3α), Long non-coding RNAs (HAS2-AS1, HIF1-AS1), Biomarkers, Immunothrombosis, Inflammation

## Abstract

The clinical course of COVID-19 ranges from mild symptoms to severe complications, and common laboratory markers such as D-dimer, ferritin, interleukin-6 (IL-6), and C-reactive protein (CRP) often do not accurately predict which patients will develop severe disease. In this study, we reviewed current literature and analyzed additional data to assess emerging biomarkers that may help identify high-risk cases earlier. These include circulating cell-free DNA (cfDNA) produced during neutrophil extracellular trap formation (NETosis), hyaluronic acid (HA), hypoxia-inducible factor (HIF) isoforms, and related long non-coding RNAs such as HAS2-AS1 and HIF1-AS1. Increased levels of cfDNA/NETs, HA, and elevated expression of HIF isoforms and their lncRNAs are closely associated with key features of severe COVID-19, including immune-related blood clotting, low oxygen levels, vascular damage, and chronic inflammation. These biomarkers show promise for use in risk assessment tools that could support earlier clinical decisions and improve outcomes in patients with COVID-19.

## Introduction

The COVID-19 pandemic, caused by the SARS-CoV-2 virus, has underscored the need for reliable biomarkers that can help predict the risk of severe disease. Although considerable progress has been made in understanding the underlying mechanisms of COVID-19, commonly used laboratory indicators such as D-dimer, ferritin, interleukin-6 (IL-6), and C-reactive protein (CRP) often fall short in accurately identifying patients who are likely to experience critical complications. The unpredictable nature of disease severity—even among patients with similar clinical profiles—suggests that additional molecular pathways may be involved in driving progression to severe illness [1, 2].

Recent research has focused on a set of less conventional biomarkers that appear to play important roles in the development of severe COVID-19. These include circulating cell-free DNA (cfDNA) released during neutrophil extracellular trap (NET) formation, hyaluronic acid (HA), hypoxia-inducible factors (HIFs), and long non-coding RNAs (lncRNAs) such as HAS2-AS1 and HIF1-AS1. These molecules are involved in processes strongly associated with severe COVID-19, such as disrupted oxygen balance, damage to the vascular system, and uncontrolled inflammation and clot formation.

This article reviews available data on these markers and considers their potential use in identifying patients more likely to develop serious complications.

## Immune dysregulation in severe COVID-19

Under ideal circumstances, the immune system provides a balanced response that resists invading microorganisms while allowing the host to survive the infection [3, 4]. For many infections, coordinated production of stable circulating levels of pro-inflammatory cytokines is a natural outcome of appropriate innate and adaptive immune responses [5–7]. These responses are effective at controlling pathogens without causing harm to the host [8, 9]. However, these defense mechanisms have limits, and excessive or prolonged cytokine production can deplete regulatory capacities [10–12].

Genetic variation plays an important role in shaping individual immune responses, affecting the likelihood of either an overactive inflammatory reaction or inadequate immune defense [13–15]. Differences in genes that control cytokine production and function can disrupt the balance between pro- and anti-inflammatory pathways [16–18]. In cases of excessive cytokine production, targeted pharmacological agents [19, 20]—such as interleukin-6 or tumor necrosis factor inhibitors—are employed to modulate the inflammatory response [21–23]. The use of therapeutics helps prevent the development of cytokine storms and reduces tissue damage [24–26]. Other therapeutic agents also have demonstrated efficacy in enhancing antiviral defenses and modulating hyperinflammatory states [27–30]. The composition and diversity of the gut microbiota have also been shown to influence immune homeostasis and the course of infectious diseases, particularly in patients with comorbidities [31–34]. Recent studies also emphasize the importance of personalized treatment strategies based on the patient’s genetic profile to achieve optimal control of the immune response [35, 36].

There is no definitive answer as to why COVID-19 has caused such a high rate of severe cases compared to other viral infections. However, it is hypothesized that the increased expression of the angiotensin-converting enzyme 2 (ACE2) receptor with aging and comorbidities—serving as the entry point for SARS-CoV-2 [37, 38]—along with impaired antiviral adaptive immunity, particularly the interferon (IFN) response, may be key factors. This impaired response could lead to prolonged viral persistence and unregulated inflammatory reactions, similar to what has been observed with other zoonotic coronaviruses such as SARS and MERS [39, 40].

It is therefore suggested that SARS-CoV-2 infection induces a significant imbalance in the innate immune response, characterized by suppression of peripheral innate immunity alongside activation of pro-inflammatory pathways [41]. In severe and critical COVID-19 patients, a distinct phenotype has been observed: markedly reduced type I interferon responses associated with persistent viremia and exacerbated inflammation [42].

## NETosis and extracellular DNA as prognostic markers of severe COVID-19

Neutrophil activation is one of the earliest immune responses to infection. Activated neutrophils can form neutrophil extracellular traps (NETs), which contain extracellular DNA (ecDNA), histones, and enzymes such as myeloperoxidase (MPO). Under normal conditions, this serves to localize pathogens. However, excessive or uncontrolled NET formation induces inflammation, endothelial damage, and thrombosis [43–46].

Our study demonstrated that patients with COVID-19 who later developed severe disease had plasma ecDNA levels 3.4 times higher at admission than healthy individuals, with statistically significant differences compared to patients with mild disease [47]. An ecDNA concentration > 4297 ng/mL correlated with an increased risk of hypoxia, cytokine storm, and the need for intensive care. Correlation analysis confirmed a moderate association between NETosis and ecDNA levels, while the ROC curve highlighted the efficacy of ecDNA as a prognostic marker of disease severity [47]. We also measured MPO, a marker of NET formation, and showed a clear increase in MPO levels correlating with disease severity—fourfold higher in severe cases compared to mild [47] (Table 1).

**Table 1:** Summary of key studies on prognostic markers in COVID-19

Author (year)	Country	Methods	Study Population	Primary aim	Key Findings
Zhu L. et al. (2020)	China	Retrospective cohort study	7337 patients with COVID-19 and type 2 diabetes	To assess the impact of glycemic control on the course of COVID-19	Well-controlled blood glucose was associated with lower mortality and fewer complications compared to poorly controlled glucose.
Ackermann M. et al. (2021)	Germany	Review article	Patients with severe COVID-19	To investigate the role of NETs in the pathogenesis of COVID-19	NETs contribute to immunothrombosis, organ damage, and inflammation, especially in severe cases.
Shah C. et al. (2020)	USA	Retrospective observational	Hospitalized COVID-19 patients	To identify mortality-associated risk factors	Age, ICU admission, and elevated CRP and D-dimer levels were associated with increased mortality.
Hadjadj J. et al. (2020)	France	Cohort study	50 COVID-19 patients (mild, moderate, and severe cases)	To evaluate type I interferon response and inflammation in COVID-19 patients of varying severity	Severe patients showed impaired type I interferon responses and increased inflammation, correlating with worse outcomes.
Janiuk K. et al. (2021)	Poland	Review article	Not specified (literature review)	To analyze the role of NETs in COVID-19	NETs play a significant role in the development of immunothrombosis, associated with severe disease progression.
Dubrovskyi E.I. et al. (2023)	Ukraine	Clinical observation	COVID-19 patients with type 2 diabetes and obesity (93 patients, 10 controls)	To investigate cfDNA levels as a marker of disease progression	Elevated cfDNA and MPO levels correlated with more severe clinical outcomes, indicating potential as predictive biomarkers.
Middleton E.A. et al. (2020)	USA	Cohort study	COVID-19 patients with ARDS (*n* ≈ 50)	To evaluate the role of NETs in immunothrombosis during acute respiratory distress syndrome (ARDS)	NETs promoted thrombosis in the pulmonary microcirculation; high NET levels were associated with increased ARDS severity.
Zuo Y. et al. (2020)	USA	Cohort study	80 COVID-19 patients, 30 control individuals	To assess NET levels and their association with clinical status	COVID-19 patients had significantly elevated levels of cfDNA, MPO-DNA, and H3Cit, which increased with disease severity.
Andargie T.E. et al. (2021)	USA	Cohort study	Over 100 patients	To evaluate cfDNA as a marker of tissue damage and mortality risk	cfDNA correlated with organ damage severity and mortality; also induced tissue injury.
Huckriede J. et al. (2021)	Netherlands	Cohort study	126 individuals (COVID-19 patients, controls, and donors)	To assess the prognostic value of NETosis and DAMPs in severe cases	Dynamic levels of cfDNA and GAS6 were linked to ventilator-free days and mortality; significant correlation with clinical parameters observed.
Hammad R. et al. (2021)	Egypt	Cohort study	105 COVID-19 patients	To evaluate cfDNA levels, lymphocyte subpopulations, and neutrophil/lymphocyte ratio in relation to COVID-19 severity	Elevated cfDNA and altered immune cell ratios correlated with severe disease progression.
Stawski R. et al. (2022)	Poland	Review	Not specified	To assess cfDNA as a diagnostic and prognostic marker in COVID-19	cfDNA may serve as a biomarker for disease severity, organ injury, and inflammation.
Hellman U. et al. (2020)	Sweden	Histological study	Lung samples from patients with severe COVID-19	To determine the presence of hyaluronic acid in lung alveoli	Hyaluronic acid was found at high concentrations in the lungs of severely ill patients; a potential therapeutic target.
Zhao F. et al. (2022)	USA	Pre-clinical study	Animal models	To evaluate hyaluronic acid as a target in ARDS using radiolabeling techniques	Hyaluronic acid may be a target for diagnosis and treatment of lung injury in ARDS.
Dubrovskyi E. et al. (2024)	Ukraine	Clinical study	78 COVID-19 patients	To compare the prognostic value of HAS2-AS1 lncRNA versus plasma hyaluronic acid	High HAS2-AS1 levels better predicted clinical outcomes than plasma HA levels.
Ding M. et al. (2020)	China	Correlation analysis	32 COVID-19 patients	To analyze the relationship between severity and clinical prognosis in COVID-19	Severe disease was associated with elevated biomarkers, such as hyaluronic acid and type III procollagen; these parameters identified critical patients at admission and were linked to worse prognosis.
Hellman U. et al. (2024)	Sweden	Prospective cohort study	COVID-19 patients (exact number not specified)	To assess in vitro and in vivo hyaluronic acid levels and their association with lung injury	Elevated hyaluronic acid levels correlated with prolonged pulmonary complications.
Zhao F. et al. (2022)	USA	Pre-clinical study	Animal models	To evaluate the use of labeled hyaluronic acid for detecting lung injury in ARDS	Feasibility of diagnosing lung inflammation and injury using labeled hyaluronic acid was confirmed.
Rosser J.I. et al. (2022)	USA	Clinical study	Human volunteers	To assess hymecromone efficacy in reducing hyaluronic acid levels	Hymecromone intake significantly reduced HA levels—potential therapy for COVID-19.
Dubrovskyi E.I. et al. (2024)	Ukraine	Conference presentation and abstract publication	36 patients with diabetes/obesity and 7 healthy controls	To assess HIF-3α expression in leukocytes as a prognostic marker in COVID-19	High HIF-3α expression in leukocytes was associated with unfavorable clinical outcomes.
Dubrovskyi E. et al. (2024)	Ukraine	Clinical study	36 patients with diabetes/obesity and 7 healthy controls	To investigate HIF-1α and HIF1A-AS1 as markers of severe COVID-19	High expression of HIF-1α and HIF1A-AS1 in buffy coat correlated with worse clinical outcomes and prognosis.
Tian M. et al. (2021)	China	Cellular and in vivo study	Mouse models and cell lines	To investigate the role of HIF-1α in SARS-CoV-2 pathogenesis	HIF-1α promoted SARS-CoV-2 infection and exacerbated inflammation; a potential therapeutic target.
Cichon I. et al. (2021)	Poland	Experimental study	Obesity and systemic inflammation mouse models	To study NET metabolic pathways in the context of obesity and LPS	NETosis metabolic pathways differ in obesity, which is relevant for COVID-19 pathogenesis.
Branitzki-Heinemann K. et al. (2016)	Germany	Experimental	Human neutrophils	To study the effect of hypoxia on NET formation	Hypoxia enhances NET formation—relevant to COVID-19 pathogenesis.

These findings are corroborated by multiple studies. Liu B et al. was among the first to suggest that fatal COVID-19 complications result more from vascular injury caused by extracellular DNA and cytokine storms than by the virus itself [48]. Sorensen and Borregaard found that NETs, through ecDNA production, contribute more to autoimmune dysregulation and thrombosis than to protective immune responses [43].

Middleton et al., Zuo et al., Andargie et al., Huckriede et al., and others have all highlighted the association between elevated cfDNA, NET-induced thrombosis, and poor COVID-19 outcomes [49–52]. In a large study, Andargie et al. found that plasma cfDNA levels during the first two days of hospitalization were 4.5 times higher in patients who ultimately died of COVID-19 compared to survivors, identifying cfDNA profiles as predictive markers of disease [51]. Notably, mean cfDNA levels were 4.7 and 67 times higher in COVID-19 patients compared to those with influenza and rhinovirus infections, respectively. The authors emphasized that neutrophils were the main contributors to elevated cfDNA, especially in patients requiring intensive care or who died [51].

Independent groups led by Zuo et al. and Hammad et al. also proposed that NET production and plasma cfDNA levels may serve as predictors of disease severity and clinical progression [50–53]. In a comprehensive analysis, Huckriede et al. examined the dynamics and significance of various markers in COVID-19, including cfDNA, neutrophil elastase, and histones, which he identified as reflective of organ dysfunction in severe disease [52]. Huckriede also showed that cfDNA levels correspond with lung function in ICU patients on invasive mechanical ventilation, and that cfDNA levels dynamically change in relation to disease progression [52]. He identified cfDNA as a DAMP capable of activating TLRs—a view shared by Stawski et al., who also concluded that extracellular DNA is a strong marker of COVID-19 complications and an indicator of patient health and risk [54].

A dedicated systematic review in 2022 compiled evidence from 10 studies (810 patients) on NETs in COVID-19 and found a strong association with disease severity. Markers of NET formation– such as myeloperoxidase-DNA complexes, citrullinated histone H3, and cfDNA– were significantly elevated in patients with severe or critical COVID-19 compared to mild cases or healthy controls. Several of the included studies reported that high levels of NET components at admission (or a failure to clear NETs over time) were predictive of poor outcomes, including the need for mechanical ventilation and in-hospital mortality [55].

In summary, the total body of literature confirm that plasma extracellular DNA is not only a reliable marker of COVID-19 severity, strongly correlated with disease progression, but also an important pathogenic factor in the disease course.

The cost-effectiveness of assessing prognostic markers varies considerably. Although our findings on neutrophil responses in COVID-19 patients were striking—for example, neutrophils from stable COVID-19 patients produced four times more NETs than those from healthy controls, and upon PMA stimulation, NETosis levels doubled compared to controls [47]—measuring NETosis is technically complex, time-consuming, and expensive. Therefore, it is not practical for widespread clinical use.

In contrast, the assessment of plasma extracellular DNA is technically simpler and more accessible. Our data and those from multiple research groups show that cfDNA measurement is more informative. It can be assessed using various laboratory techniques such as PCR or spectrofluorometry, making it feasible for use in routine clinical settings upon hospital admission.

A dedicated systematic review in 2022 compiled evidence from 10 studies (810 patients) on NETs in COVID-19 and found a strong association with disease severity. Markers of NET formation– such as myeloperoxidase-DNA complexes, citrullinated histone H3, and cfDNA– were significantly elevated in patients with severe or critical COVID-19 compared to mild cases or healthy controls. Several of the included studies reported that high levels of NET components at admission (or a failure to clear NETs over time) were predictive of poor outcomes, including the need for mechanical ventilation and in-hospital mortality. For example, one cohort noted that patients with the smallest decline in plasma cfDNA over the first week required the longest ventilatory support, and persistently high NET levels portended higher mortality risk.

## Hyaluronic acid and long Non-Coding RNA HAS2-AS1 as early markers of hypoxia

One of the earliest manifestations of severe COVID-19 is interstitial pulmonary edema, visualized as “ground-glass opacities” on CT imaging [56–59]. Accumulation of hyaluronic acid (HA) in lung tissues is considered a key mechanism in this process. HA is a glycosaminoglycan capable of binding large volumes of water, forming a gel-like substance that impairs gas exchange and exacerbates hypoxia [60, 61].

Our experimental data demonstrated a clear correlation between plasma HA levels in COVID-19 patients and disease progression, even before the onset of clinical complications. In patients with severe COVID-19, plasma HA levels were twice as high as those in the mild and control groups, and 1.4 times higher than in the moderate group. These levels strongly correlated with the risk of clinical hypoxia and respiratory failure [62]. Even more informative was the expression level of the long non-coding RNA HAS2-AS1, which regulates the stability of HAS2 mRNA (hyaluronic acid synthase 2).

Compared to HA plasma levels, HAS2-AS1 expression in leukocytes at the time of hospital admission proved to be a more reliable and significant prognostic marker. It clearly distinguished patients with subsequent mild, moderate, or severe courses of disease. In the moderate group, HAS2-AS1 expression increased by 7.7-fold (*p* < 0.05), and by 22.6-fold (*p* < 0.05) in the severe group compared to the mild group [62].

The prognostic significance of HAS2-AS1 is further supported by a very high effect size (ω² = 0.33), indicating that HAS2-AS1 has a strong influence on COVID-19 outcomes in the general population. ROC analysis (sensitivity 0.88, specificity 0.80) confirms that HAS2-AS1 is a highly effective tool for individual prediction of disease severity. Our identified threshold value of 61.8 a.u. suggests that patients with HAS2-AS1 expression above this level are at significantly increased risk (88%) of complications. We propose that HAS2-AS1 can be utilized to build stratified risk models in combination with plasma HA levels [62]. Although the regulation and expression of HAS2-AS1 in the context of COVID-19 remain underexplored, other researchers have also reported supporting data. Elevated HA in plasma and respiratory secretions of severe COVID-19 patients strongly correlates with disease progression.

In 2023, Borrmann et al. showed that plasma HA levels in hospitalized COVID-19 pneumonia patients were significantly higher at admission in those with severe disease compared to early recoverers. The authors demonstrated that circulating glycocalyx components during SARS-CoV-2 infection act as biologically active markers of microvascular damage, adversely affecting signaling pathways in immune and endothelial cells, thereby promoting inflammation and microthrombosis [63].

Also, in 2023, Huang described the pathological role of the extracellular matrix, including HA, in COVID-19 development, emphasizing its value as a prognostic marker [64]. Similarly, Ding et al. identified HA as an early warning indicator of poor prognosis in severe COVID-19 patients [65].

In 2024, Hellman, one of the first to hypothesize the link between HA and interstitial lung edema [60], used 3D lung morphology analysis to demonstrate the presence of fragmented HA in severe COVID-19 and correlated systemic HA levels with post-recovery pulmonary diffusion capacity. Hellman further suggested that HA-targeted therapy may be relevant not only for SARS-CoV-2 infection but also for other pulmonary infections [66].

Zhao described COVID-19-associated ARDS through the lens of HA and NETosis interaction: high HA levels combined with NETs form gel-like structures accumulating in alveoli, causing severe edema and impairing gas exchange [61].

NETs and HA may synergize with pro-inflammatory cytokines such as IL-6 and TNF-α, creating a vicious cycle—elevated cytokine levels stimulate NET and HA production, which in turn enhances inflammation and edema. This feedback loop may significantly worsen COVID-19 outcomes and drive complications [63]. Viewing HA not only as a pathogenic factor or disease severity indicator, but also as a potential therapeutic target opens new intervention opportunities.

**Fig. 1:**
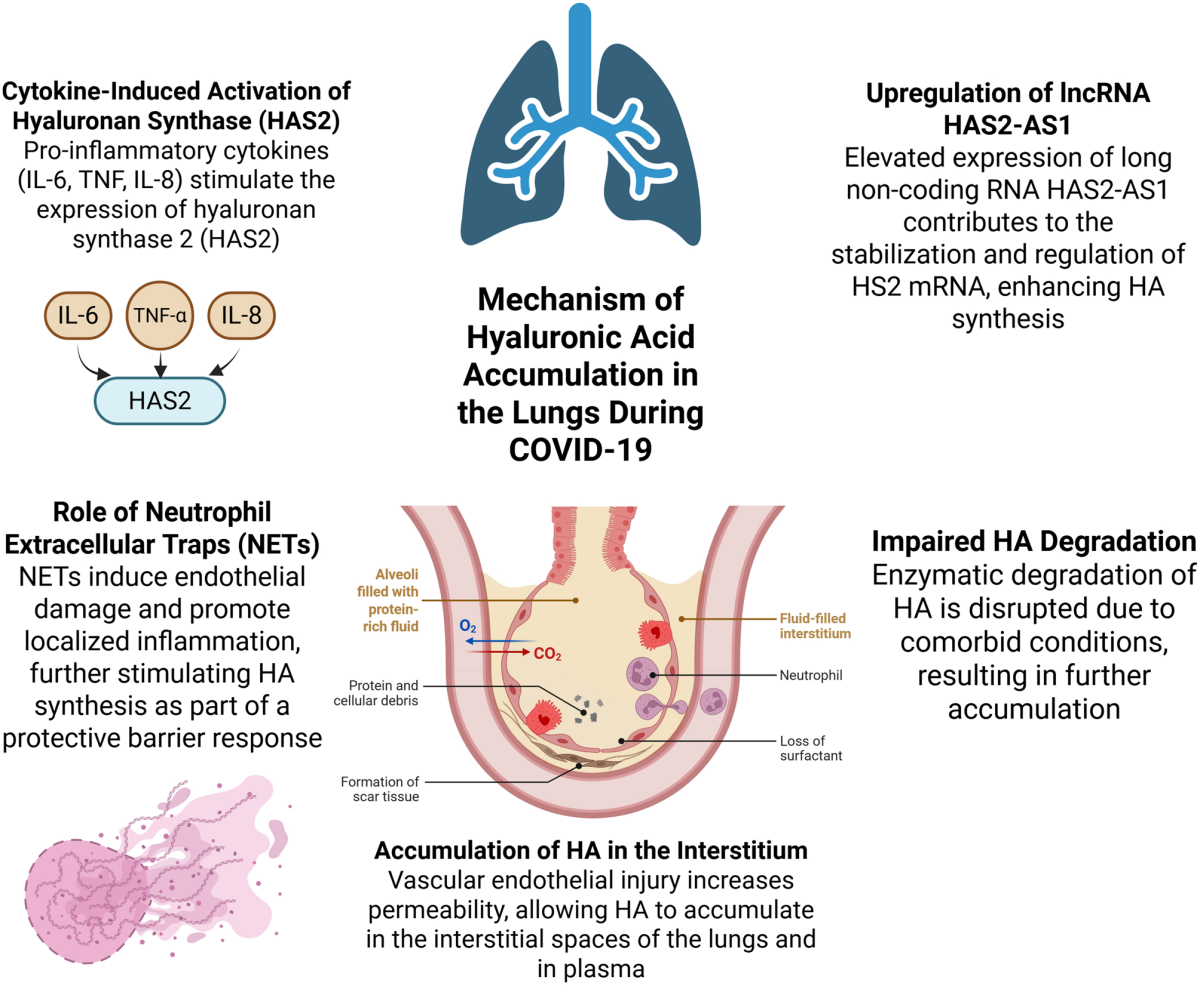
Mechanism of Hyaluronic acid accumulation in the lungs during COVID-19. Cytokines such as IL-6, TNF, and IL-1β stimulate the activity of hyaluronan synthases (HAS2), which are responsible for the production of hyaluronic acid (HA). In parallel, hypoxia and the activation of hypoxia-inducible factors HIF-1α and HIF-2α promote the transcription of genes involved in HA synthesis, including HAS2 and the expression of long non-coding RNA HAS2-AS1. Additionally, neutrophil extracellular traps (NETs) contribute to the upregulation of HA synthesis. NETs induce endothelial damage and enhance local inflammation, which further stimulates HA production as part of a protective barrier response. HA accumulation in the lung interstitium is facilitated by in-creased vascular permeability, secondary to endothelial injury, allowing HA to build up both in the interstitial lung spaces and in the plasma. It is also important to note that impaired degradation of HA plays a significant role, as hyaluronidase activity may be diminished in the context of comorbid conditions

Several studies have shown that hymecromone (4-Methylumbelliferone, 4-MU), an FDA-approved antispasmodic agent for biliary tract disorders, directly inhibits HA synthesis [67]. It has been proven to reduce HA levels in serum and sputum of healthy individuals [68]. A recent clinical trial showed that hymecromone effectively suppresses COVID-19 progression and warrants further investigation [69]. The role of HA in interstitial pulmonary edema formation in severe COVID-19 is schematically illustrated in Fig. 1.

## Transcription factor HIF and long Non-Coding RNA HIF1-AS1 in the context of COVID-19

Hypoxia is a central element in the pathogenesis of severe COVID-19. Cellular adaptation to hypoxic conditions is mediated by the activation of hypoxia-inducible factors (HIFs), among which the best-studied isoforms are HIF-1α, HIF-2α, and HIF-3α. Under normal conditions, HIFs regulate the expression of hundreds of genes involved in angiogenesis, metabolism, cell survival, and immune responses [70].

In our study, we assessed the expression of various HIF isoforms (HIF-1α, HIF-2α, HIF-3α) and found that the expression of all subunits significantly increased in COVID-19 patients. Moreover, expression levels at disease onset directly correlated with subsequent disease severity. In patients with mild and moderate COVID-19, HIF-2α expression increased the most (6-fold and 10.6-fold relative to controls), followed by HIF-1α (4.3-fold and 8.2-fold), and to a lesser extent, HIF-3α (6.2-fold and 7.1-fold). In severe cases, HIF-2α showed a 17.7-fold increase, while HIF-1α and especially HIF-3α expression rose sharply (21.6-fold and 33.7-fold, respectively). These findings suggest extensive transactivation of hypoxia-responsive genes in severe patients, potentially fueling cytokine storm and inflammation, despite the supposed regulatory role of HIF-3α.

We also examined the expression of the long non-coding RNA HIF1-AS1, which modulates HIF-1α activity. In patients with moderate disease, HIF1-AS1 expression increased 5-fold, and nearly 10-fold in severe cases, compared to mild cases. ROC analysis showed 100% specificity and 73% sensitivity at a threshold value of 277.85 a.u [71].

Simultaneous measurement of HIF-1α, HIF-3α, and HIF1-AS1 expression may serve as a reliable model for early prediction of COVID-19 severity, especially in patients with comorbidities such as diabetes and obesity. Our literature review confirms the complex and sometimes contradictory role of HIFs in the pathogenesis of infectious and metabolic diseases [70, 72, 73].

In COVID-19, HIF activation enhances neutrophil survival, metabolic activity, and NET formation [74, 75]. Some studies indicate that excessive HIF activation promotes cytokine storm, endothelial injury, and ARDS progression [76, 77].

However, the distinct roles of different HIF subunits in COVID-19 remain poorly understood. Our findings highlight the importance of evaluating the relative expression of HIF subunits. In severe COVID-19, we observed significant imbalances in HIF-1α/HIF-3α and HIF-2α/HIF-3α ratios, suggesting a breakdown in hypoxic adaptation and the onset of pathological inflammation.

This precludes a definitive conclusion. We hypothesize that dysregulated adaptation and HIF cross-regulation underlie decompensation and progression to severe hypoxia in COVID-19. The theoretically antagonistic role of HIF-3α toward HIF-1α (whose expression increased 4.7-fold compared to moderate cases and 33-fold compared to controls) was expected to inhibit HIF-1α activity and reduce NET formation. However, our data suggest the opposite: elevated HIF-1α, HIF-2α, and HIF1-AS1 expression in severe cases not only fails to prevent hypoxic tissue injury but may promote neutrophil survival and uncontrolled NET formation, further exacerbating tissue damage and thrombosis.

HIF-3α acts both as an independent transcription factor and as a dominant-negative regulator, forming dimers with other alpha subunits and preventing their binding to beta subunits and nuclear translocation [78–80]. Huang et al. also describe the balancing regulatory role of HIF-3α, although they refrain from concluding that its dominant-negative function is absent. Existing data suggest HIF-2α and HIF-3α have distinct target genes [81, 82].

Our results showed that hypoxia induces HIF-3α expression more rapidly than HIF-1α in cardiac ventricles, with levels increasing proportionally with hypoxia severity [83], emphasizing the regulatory significance of HIF-3α. Drevytska et al. proposed that alternative splicing variants of HIF-3α may modulate HIF-1α and HIF-2α function. Furthermore, HIF-1α and HIF-2α activation appears to transcriptionally induce HIF-3α as a target gene [84].

Nonetheless, our data indicate that this regulatory system fails in many COVID-19 patients and does not prevent decompensation or progression to severe disease. HIF-3α dysregulation may impair hypoxic adaptation. Some studies show that HIF-3α suppression improves physical endurance and enhances hypoxic adaptation [85].

## The interconnection between transcription factor HIF, hyaluronic acid, and neutrophil extracellular trap formation

HIF-1α regulates the expression of genes that promote cell survival, such as glycolytic enzymes. Its inhibition or deletion in macrophages leads to a sharp drop in ATP levels, impairing their motility and bactericidal activity [85]. A similar phenomenon is observed in neutrophils, where increased HIF-1α expression causes a metabolic shift toward glycolysis, the primary ATP source for these cells [74].

Given that neutrophils play a key role in hyperinflammation and immune response activation through NETosis, it has been shown that HIF-1α expression correlates with NET release upon bacterial lipopolysaccharide stimulation, and that HIF-1α inhibition completely prevents NET formation [74].

Another study demonstrated that stabilization of HIF-1α enhances NET release, whereas pharmacological or genetic HIF-1α knockdown results in reduced NET formation and decreased extracellular bacterial killing [86]. The same study cites findings that neutrophil incubation under severe hypoxia abolishes PMA-induced NET formation [87], possibly due to hypoxia-induced suppression of ROS production—a key element for NETosis [88]. However, this could also be attributed to an overall decrease in cell reactivity due to energy depletion under hypoxic conditions.

Nevertheless, it can be hypothesized that the HIF response to acute hypoxia, while potentially protective, also promotes neutrophil survival and enhances NET formation. As with other immune cells, maintaining a delicate balance between neutrophil activation during early inflammation and subsequent resolution is essential to prevent tissue damage.

The interplay between NETs, hyaluronic acid (HA), and the transcription factor HIF plays a critical role in the host response to COVID-19-associated hypoxia. HIF is a central regulator of cellular adaptation to low oxygen levels, a hallmark of lung pathology in COVID-19. Upon hypoxia, HIF activation supports neutrophil survival and stimulates NET formation. While this aids in pathogen clearance, in COVID-19, where hypoxia is persistent and systemic, excessive HIF activation may lead to heightened NETosis and extracellular DNA release, fueling inflammation.

HIF also affects cells responsible for HA synthesis, such as fibroblasts and endothelial cells. Under hypoxic conditions, HIF induces the expression of hyaluronan synthase enzymes (HAS1, HAS2), leading to HA accumulation in tissues. This results in the formation of gel-like matrices that promote edema and impair gas exchange, exacerbating respiratory dysfunction in severe COVID-19.

Severe COVID-19 thus appears to be driven by a vicious cycle: HIF activation promotes neutrophil activation and NETosis, causing tissue damage and inflammation, which in turn stimulates HA production as a response to injury. HA, combined with extracellular DNA from NETs, creates a high-viscosity environment that impairs ventilation and contributes to pulmonary edema, characteristic of acute respiratory distress syndrome (ARDS) in critical COVID-19 cases.

**Fig. 2:**
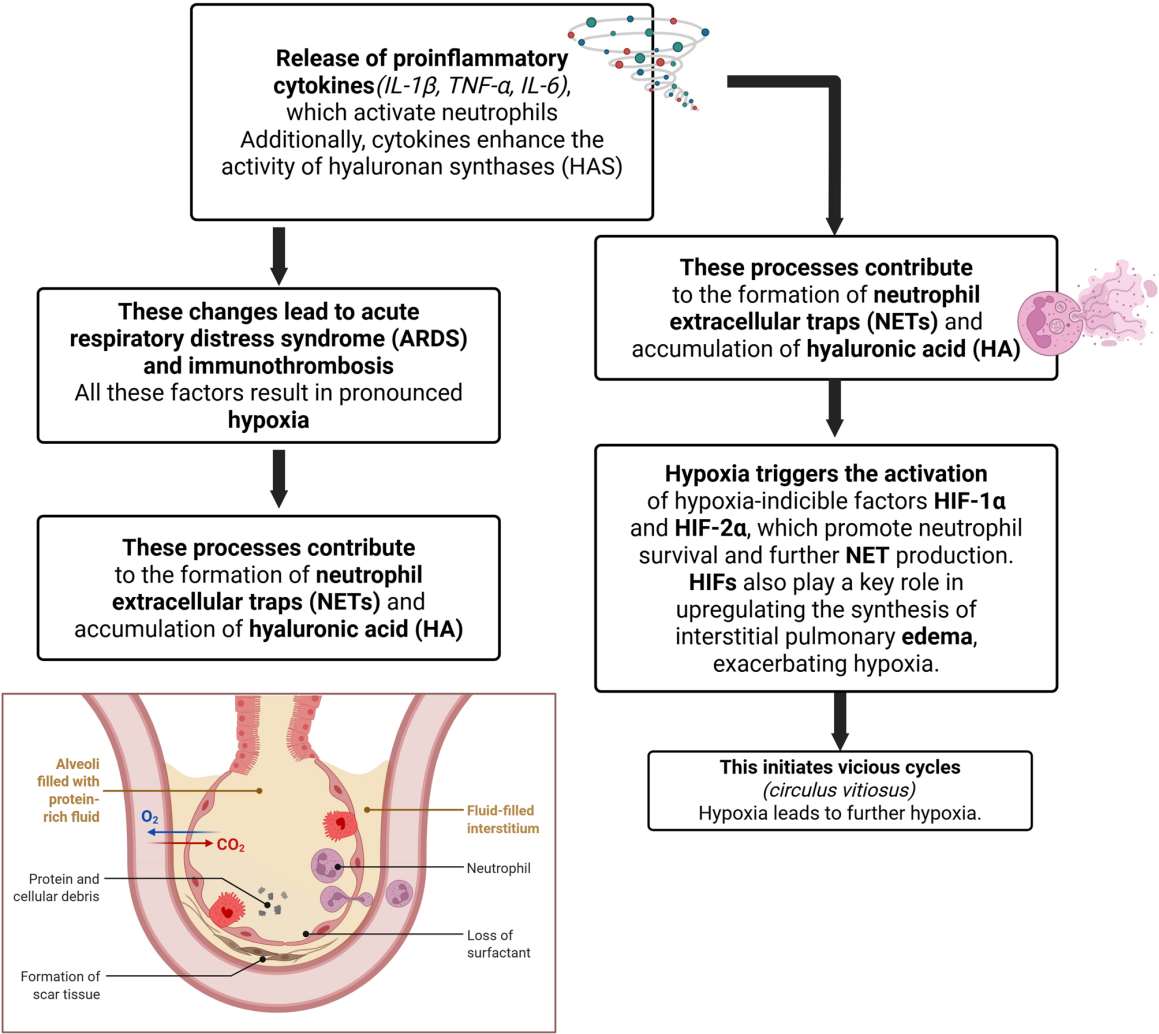
Scheme about summary of pathogenetic mechanisms of severe COVID-19. The release of proinflammatory cytokines activates neutrophils and enhances the activity of hyaluronan synthases (HAS). This promotes the formation of neutrophil extracellular traps (NETs) and the accumulation of hyaluronic acid (HA) in the interstitial space. NETs induce endothelial injury and contribute to microthrombosis in pulmonary vessels, while hyaluronic acid facilitates the development of hypoxia. Hypoxia triggers the activation of hypoxia-inducible factors HIF-1α and HIF-2α, which promote neutrophil survival and further NET production. HIFs also play a key role in amplifying the synthesis of hyaluronic acid. Hyaluronic acid further contributes to the development of interstitial pulmonary edema, thereby exacerbating hypoxia. This establishes two vicious cycles (circulus vitiosus). Hypoxia leads to further hypoxia, while HIFs inhibit apoptosis, promoting the survival of SARS-CoV-2-infected cells and further enhancing NETosis by neutrophils

Understanding this complex interaction opens avenues for novel therapeutic strategies targeting specific points in this pathological cascade—particularly maintaining HIF regulation to limit NET formation and HA production. A schematic overview of the pathophysiological mechanisms underlying severe COVID-19, integrating our findings with current literature, is presented in Fig. 2.

## Combined prognostic models: new strategies for risk stratification

Plasma extracellular free DNA is an important laboratory indicator that enables early screening of SARS-CoV-2 patients to predict disease severity, oxygen deficiency, and the potential for early therapeutic intervention.

Its measurement is justified by its proven involvement in three key pathogenic processes: thrombosis, autoimmune injury, and activation of immune cell membrane receptors.

The methodology for assessing extracellular DNA in plasma is simple and accessible. As shown in our study, it yields informative results and can be implemented in clinical practice using techniques such as PCR or spectrofluorometry upon patient admission.

Implementing assays for long non-coding RNAs HAS2-AS1 and HIF1A-AS1 in clinical settings may offer valuable prognostic information and aid in effective patient triage at the outpatient level or upon early hospitalization. These tests are also straightforward and widely accessible.

The ratio between different HIF isoforms during COVID-19 progression holds significant prognostic value. It complements the isolated measurement of HIF expression levels and highlights the role of HIF-3 in the hypoxic response. Although studies on HIF-3α regulation in COVID-19 are still in their early stages and require further exploration, they offer potential for developing novel therapeutic strategies for severe disease.

Given the limited prognostic accuracy of conventional clinical and laboratory parameters, using combined models incorporating the aforementioned biomarkers is a promising direction in personalized COVID-19 diagnostics. Based on our findings, we propose a multicomponent model that includes: Plasma extracellular DNA level (> 4297 ng/mL); Myeloperoxidase concentration as an indicator of NET activity; Expression levels of HAS2-AS1 and HIF1-AS1 in leukocytes; HIF-1α/HIF-3α ratio as a marker of hypoxic response balance.

This biomarker combination enables early identification of patients at high risk of complications—even before clinical hypoxia develops—and facilitates risk stratification that considers existing metabolic disorders. Such models can support the development of early triage algorithms during pan-demics and healthcare system overloads, allowing for optimal resource allocation to patients with poor prognoses.

Ultimately, implementing these findings in clinical practice could reduce mortality from severe COVID-19 and other respiratory illnesses associated with ARDS, while enhancing the efficiency of healthcare resource use.

The study of COVID-19 has significantly advanced our understanding of fundamental biological processes that were previously underappreciated. The realization that the immune system’s aggressive response—rather than the virus itself—can be critically harmful has reshaped our perception of viral diseases and transformed treatment strategies.

## Conclusion

The identification of reliable biomarkers remains critical for early risk assessment and improved clinical management of patients with COVID-19. Our findings demonstrate that markers such as plasma extracellular DNA, myeloperoxidase levels, hyaluronic acid, and the expression of long non-coding RNAs (HAS2-AS1 and HIF1-AS1), as well as hypoxia-inducible factors, are closely linked to disease severity and may offer valuable prognostic information.

These biomarkers reflect core pathological features of severe COVID-19, including immune dysregulation, endothelial injury, hypoxia, and thrombotic complications. In particular, the combined use of these indicators—rather than relying on any single parameter—enhances predictive accuracy and supports more effective clinical decision-making.

Measuring these biomarkers early in the course of illness may help identify patients at risk of complications, particularly those with existing health problems. As research continues, they may prove useful in adjusting treatment plans to better match the needs of individual patients with COVID-19 and other respiratory infections.

## Data Availability

No datasets were generated or analysed during the current study.
